# Progranulin Preserves Autophagy Flux and Mitochondrial Function in Rat Cortical Neurons Under High Glucose Stress

**DOI:** 10.3389/fncel.2022.874258

**Published:** 2022-07-08

**Authors:** Cass Dedert, Vandana Mishra, Geetika Aggarwal, Andrew D. Nguyen, Fenglian Xu

**Affiliations:** ^1^Department of Biology, College of Arts and Sciences, Saint Louis University, St. Louis, MO, United States; ^2^Henry and Amelia Nasrallah Center for Neuroscience, Saint Louis University, St. Louis, MO, United States; ^3^Department of Pharmacology and Physiology, School of Medicine, Saint Louis University, St. Louis, MO, United States; ^4^Department of Internal Medicine, Division of Geriatric Medicine, School of Medicine, Saint Louis University, St. Louis, MO, United States

**Keywords:** autophagy, neurodegeneration, progranulin, hyperglycemia, diabetes, cortical neurons

## Abstract

Chronic hyperglycemia in type II diabetes results in impaired autophagy function, accumulation of protein aggregates, and neurodegeneration. However, little is known about how to preserve autophagy function under hyperglycemic conditions. In this study, we tested whether progranulin (PGRN), a neurotrophic factor required for proper lysosome function, can restore autophagy function in neurons under high-glucose stress. We cultured primary cortical neurons derived from E18 Sprague-Dawley rat pups to maturity at 10 days *in vitro* (DIV) before incubation in high glucose medium and PGRN for 24-72 h before testing for autophagy flux, protein turnover, and mitochondrial function. We found that although PGRN by itself did not upregulate autophagy, it attenuated impairments in autophagy seen under high-glucose conditions. Additionally, buildup of the autophagosome marker light chain 3B (LC3B) and lysosome marker lysosome-associated membrane protein 2A (LAMP2A) changed in both neurons and astrocytes, indicating a possible role for glia in autophagy flux. Protein turnover, assessed by remaining advanced glycation end-product levels after a 6-h incubation, was preserved with PGRN treatment. Mitochondrial activity differed by complex, although PGRN appeared to increase overall activity in high glucose. We also found that activation of extracellular signal-regulated kinase 1/2 (ERK1/2) and glycogen synthase kinase 3β (GSK3β), kinases implicated in autophagy function, increased with PGRN treatment under stress. Together, our data suggest that PGRN prevents hyperglycemia-induced decreases in autophagy by increasing autophagy flux via increased ERK1/2 kinase activity in primary rat cortical neurons.

## Introduction

Type II diabetes (T2D) is a metabolic disease characterized by chronic hyperglycemia, or elevated blood glucose. Hyperglycemia specifically contributes to pathology through several mechanisms, including pro-inflammatory signaling (Chang and Yang, 2016), accumulation of glycated proteins (Singh et al., 2014), and impairment of autophagy (Moruno et al., 2012; Mir et al., 2015). Additionally, hyperglycemia is a known risk factor for several neurodegenerative diseases such as Alzheimer’s and Parkinson’s (Sergi et al., 2019; Madhusudhanan et al., 2020). The prevalence of Parkinson’s is higher in those with diabetes compared to non-diabetics, and those with diabetes experience more severe Parkinsonian symptoms (Pagano et al., 2018). Despite the breadth of these conditions, all of them share a common underlying pathology: protein aggregates that are normally removed instead accumulate in the cells due to downregulation of autophagy (Ross and Poirier, 2004).

Autophagy is a cellular self-degradation process that is upregulated in response to a number of cell stressors, including starvation (Mizushima and Klionsky, 2007), endoplasmic reticulum stress (Ogata et al., 2006), and excessive buildup of proteins and organelles (Liu and Li, 2019). Targeted substrates are enclosed in a double-membrane vesicle called an autophagosome, which fuses with a lysosome to facilitate controlled degradation of its contents (Khandia et al., 2019). Despite its known pro-survival properties, uncontrolled autophagy leads to cell death (Denton and Kumar, 2019); because of this, the activity of this process is low under basal conditions, and the signaling pathways leading to its upregulation are tightly controlled. Nonetheless, it remains a powerful tool for maintaining cellular well-being. The importance of proper autophagy function is elevated in the nervous system due to the limited regenerative capacity and post-mitotic nature of neurons (Stavoe and Holzbaur, 2019). However, evidence indicates that the surrounding glia also contribute to neuronal health through regulation of autophagy and protein clearance (Ortiz-Rodriguez and Arevalo, 2020). Likewise, lysosomal dysfunction in astrocytes has been shown to contribute to neurodegeneration (Di Malta et al., 2012).

Progranulin (PGRN) is an endogenous neurotrophic factor expressed in high amounts in brain tissue (Nguyen et al., 2013b) that is implicated in anti-inflammatory activity in microglia (Martens et al., 2012). Furthermore, mutations in the *GRN* gene have been linked to Alzheimer’s and frontotemporal lobar dementia (FTLD), indicating a protective role against neurodegenerative disease (Baker et al., 2006; Cruts et al., 2006; Gass et al., 2006; Perry et al., 2013). FTLD is similar to other neurodegenerative diseases in that it is also characterized by buildup of protein aggregates, namely TAR DNA-binding protein 43 (TDP-43) (Cairns et al., 2007). Interestingly, PGRN is important to lysosome function, as it is trafficked to the lysosome and cleaved into granulin subunits that facilitate lysosomal function (Smith et al., 2012; Almeida et al., 2016). Accordingly, overexpression of PGRN in Alzheimer’s disease mouse models has been linked to decreased amyloid-β plaques (Minami et al., 2014; Van Kampen and Kay, 2017). These findings suggest a model in which PGRN may prevent the development of neurodegenerative pathology arising from impaired degradation of protein aggregates.

In this study, we examined the role and mechanism that high glucose plays in development of neuropathology with regards to autophagy inhibition, and the potentially protective role of PGRN. We found that autophagic activity and protein turnover were reduced in neurons incubated in high glucose conditions, with PGRN pre-treatment attenuating its harmful effects. PGRN treatment prevented pathology and reduced function due to high glucose in both cases. Mitochondrial function was affected by PGRN in cells cultured in high glucose, although the net effect varied by complex. We also observed changes in extracellular signal-regulated kinase (ERK) and glycogen synthase kinase 3-beta (GSK3β) phosphorylation in response to PGRN under high-glucose conditions.

The data we present suggest a potential role for PGRN in restoring autophagy in neurons affected by high glucose conditions, and further connect hyperglycemia and neurodegeneration through downregulation of autophagy.

## Materials and Methods

### Animals and Cell Culture

All experiments were performed on cortical neurons from the brains of E18 Sprague-Dawley rat pups, which were removed under sterile conditions according to the standard protocol approved by the Institutional Animal Care and Use Committee (IACUC), Saint Louis University, St. Louis, MO guidelines. The dissected cortices were cut into small pieces and incubated in an enzymatic solution containing 40 units of papain (Worthington Biochemical, Cat# LS003126), 2 mM CaCl_2_ (Sigma, Cat# C4901-100G), 1 mM EDTA (Sigma, Cat# E9884-100G), and 1.5 mM L-cysteine (Sigma, Cat# 168149-25G) in Neurobasal medium (Gibco, Cat# 21103-049). Tissues in solution were incubated for 30 min at 37°C, mixing every few minutes to ensure even dissolution. Tissues were then triturated through fire-polished glass pipettes, then plated on dishes coated with 2 μg/ml laminin (Sigma, Cat# 11243217001) and 100 μg/ml poly-D lysine (Sigma, Cat# P6407-5MG), and cultured in Neurobasal medium supplemented with 1X GlutaMAX (Gibco, Cat# 35050-061), 1% pen/strep (Gibco, Cat# 15140122), 2% B-27 supplement (Gibco, Cat# 17504-044), and 4% fetal bovine serum (Avantor, Cat# 97068-086). One half of the medium was changed every 3 to 4 days, and cells were grown for 10 days *in vitro* (DIV) before experimentation.

For qPCR and western blot studies in microglia, HMC3 human microglial cells (ATCC, Cat# CRL-3304) were cultured at a density of 120,000 cells/dish in 6-well plates in EMEM (Eagle’s Minimum Essential Medium) (Corning, Cat# MT10009CV) supplemented with 10% fetal bovine serum (Gibco, Cat# 26140-095), 10 U/ml penicillin, and 10 μg/ml streptomycin. Cells were cultured for 24 h, then treated with filtered glucose dissolved in autoclaved water to get a final concentration of 30 mM. An equal volume of autoclaved water was used for the control. Cells were incubated for 72 h, then checked for mRNA and protein levels.

### Treatment in Hyperglycemic Conditions and With Progranulin

For primary cortical neurons, medium was changed at DIV 10 for equivalent medium supplemented (Sigma, Cat# G6152) to reach a final glucose concentration of 100 mM (with control medium containing 25 mM glucose), similar to other *in vitro* studies exploring hyperglycemia in neuronal cultures (Chen et al., 2016; Li et al., 2017). PGRN (R&D Systems, Cat# 2557-PG050) was added to the medium at this time to achieve a final concentration of 200 ng/ml, a concentration that is similar to plasma concentrations in patients and used in previous cell culture studies (Youn et al., 2009; Zhou et al., 2019a). Cells were treated under their respective conditions for 24 or 72 h before assay testing or harvest. Status of cells was observed using a phase-contrast microscope (IX73, Olympus) and images were taken with a Retiga R1 camera (QImaging).

For protein harvest, primary neurons were washed thrice in PBS (Gibco, Cat# 10010-031), then lysed using ice-cold N-PER lysis buffer (ThermoFisher, Cat# 87792) containing Halt protease inhibitor (ThermoFisher, Cat# 1860932) and phosphatase inhibitor (ThermoFisher, Cat#78420), then scraped from plates using a cell scraper. For PGRN measurements, HMC3 cells were rinsed with PBS, then lysed in RIPA buffer containing protease inhibitors (cOmplete™, Mini, EDTA-free, Protease Inhibitor Cocktail, Roche, Cat#11836170001). In both cell types, lysates were centrifuged at 14,000 rpm for 10 min and the supernatant was collected. Protein concentration was ascertained using a BCA Protein Assay kit (ThermoFisher, Cat# 23225).

### Cell Viability Determination

Cell viability was determined using a fluorescence-based reporter dye kit (LIVE-DEAD™ Cell Imaging Kit, ThermoFisher, R37601). After treatment, cells were washed thrice with PBS, then incubated in HBSS containing 1 μM Calcein AM and 2 μM ethidium homodimer for 45 min. Images were taken using a phase-contrast microscope (IX73, Olympus) with fluorescence light source (Lambda XL, Sutter Instrument) and Retiga R1 camera (QImaging). Viability was determined by counting the number of Calcein AM-stained cells through visual observation and calculating as a ratio to total cells in each image.

### Quantitative PCR Analysis

Total RNA was isolated from cultured HMC3 cells using a RNeasy Mini kit (Qiagen, Cat#74106) with on-column DNase digestion (Qiagen, Cat#79256). RNA was reverse-transcribed to obtain cDNA using the iScript cDNA synthesis kit (Bio-Rad, Cat#1708891), and qPCR was performed using PowerUp SYBR Green Master Mix (ThermoFisher, Cat#A25777) with a Bio-Rad CFX384 Real-Time System. The primer sequences were as follows (with F for forward and R for reverse primers): human CYCLO-F, GGAGATGGCACAGGAGGAAA; human CYCLO-R, CCGTAGTGCTTCAGTTTGAAGTTCT; human GRN-F, AGGAGAACGCTACCACGGA; and human GRN-R, GGCAGCAGGTATAGCCATCTG. Results for qPCR were normalized to the housekeeping gene CYCLO and evaluated by the comparative C_*T*_ method.

### Immunoblotting

Samples were treated with Laemmli sample buffer (Bio-Rad, Cat# 1610611) containing 350 mM DTT (Bio-Rad, Cat# 1610747) and run on a pre-cast MES-SDS gel (NuPage, Cat# NP0323BOX) in a Novex Mini-Cell device (Invitrogen, Cat# EI0001). Transfer to a 0.45 μm nitrocellulose membrane (Bio-Rad, Cat# 1620115) was performed in a Mini Protean Tetra System (Bio-Rad, Cat# 1658004). For PGRN measurement, proteins were separated on SDS-PAGE Bio-Rad TGX gels and transferred onto nitrocellulose membranes using the Bio-Rad Turbo-Blot transfer system.

Membranes were blocked in TBST containing 5% milk (Bio-Rad, Cat#1706404), then blotted using primary antibodies for light chain 3B (LC3B) (E7 × 45 XP(R), CST, Cat# 43566S), lysosome-associated membrane protein 2A (LAMP2A) (Abcam, Cat# ab18528), p-ERK1/2 (CST, Cat# 4370S), ERK1/2 (CST, Cat# 4695S), phosphorylated GSK3β (CST, Cat# 9336S), GSK3β (CST, Cat# 9315S), glyceraldehyde 3-phosphate dehydrogenase (GAPDH) (CST, Cat# 2118S), PGRN (R&D Systems, Cat# AF2557), hPGRN (an anti-human PGRN linker 5 polyclonal antibody #614 that recognizes an epitope between residues 497 and 515 (Nguyen et al., 2013a)), and β-actin (CST, Cat# 3700S). All antibodies were used at a 1:1000 dilution, except for hPGRN, which was at a 1:3000 dilution. A goat anti-rabbit (Invitrogen, Cat# 31460) or donkey anti-sheep (ThermoFisher, Cat#A16041) at a 1:5000 dilution or HRP-conjugated AffiniPure goat anti-rabbit and anti-mouse antibodies (Jackson Immuno Research Labs) at a 1:10000 dilution were used for secondary antibody incubation. Western blot data were captured using an imager (ThermoFisher, iBright FL1000) after incubating the membranes in Pierce substrate (ThermoFisher, Cat#32106). hPGRN western blots were visualized using a Chemi-Doc system (Bio-Rad). Densitometric analysis was performed using ImageJ (NIH).

### Immunofluorescence of Primary Cortical Cells

Unless specified, primary cell cultures were fixed with 4% paraformaldehyde (ThermoFisher, Cat# J19943-K2) for 20 min, permeabilized with 0.3% Triton X-100 (VWR, Cat# 0694-1L) for 5 min, and blocked in PBS containing 5% goat serum (Gibco, Cat# 16210-064) for 1 h at room temperature. To visualize autophagosome and lysosome expression, antibodies for LC3B (E7 × 45 XP(R), CST, Cat# 43566S), LAMP2A (Abcam, Cat# ab18528), microtubule-associated protein 2 (MAP2) (Invitrogen, Cat# 13-1500), and glial fibrillary acidic protein (GFAP) (EMD Millipore, Cat# AB5541) were used at a 1:200 dilution in 5% goat serum. To visualize PGRN expression in microglia, a 1:100 dilution of PGRN antibody (R&D Systems, Cat# AF2557) and 1:3000 dilution of allograft inflammatory factor 1 (Iba1) antibody (Wako, Cat# 019-19741) in 5% donkey serum were used. For secondary incubation, the following antibodies were used at a 1:500 dilution in 5% goat serum: goat anti-rabbit conjugated with Alexa Fluor 568 (Invitrogen, Cat# A11036), anti-mouse conjugated with Alexa Fluor 488 (Invitrogen, Cat# A11029), and anti-chick conjugated with Alexa Fluor 488 (Abcam, Cat# ab150169). The following antibodies were used at a 1:500 dilution in 5% donkey serum: donkey anti-rabbit conjugated with Alexa Fluor 546 (Invitrogen, Cat# A10040) and donkey anti-sheep conjugated with Alexa Fluor 647 (Invitrogen, Cat# A21448). Slides were stained with DAPI (1 μg/ml) included in the mounting media (Fluoroshield, Sigma, Cat# F6507), and images were taken using a confocal microscope (Leica, TCS SP8).

Fluorescence intensity analysis was performed by selecting regions of interest (ROIs) of cell bodies, identified by staining with the neuronal marker MAP2 or astrocytic marker GFAP. The mean fluorescence intensity of each ROI was measured, and values were normalized with control equal to 1. To prevent differences in intensity due to user error, slides were viewed under the same acquisition parameters for fluorescence images.

### Advanced Glycation End-Product Degradation Assays

Cortical cells were incubated with 50 μg BSA-AGE (Cayman Chemical, Cat# 22968) for 6 h at the end of the 72-h treatment period. Protein samples were harvested and AGE detection was performed using a fluorometric assay kit (Biovision, Cat# K929-100), measuring emission at 460 nm in response to excitation at 360 nm and using BSA control as the baseline. Levels of AGEs were validated by western blot with anti-AGE antibody (Bioss, Cat# bs-1158R) at a 1:1000 dilution, normalized to GAPDH expression.

### Mitochondrial Complex Enzyme Activity Assay

Activity of ubiquinone oxidoreductase (UO), succinate dehydrogenase (SDH), and cytochrome C oxidase (COX) were tested to represent mitochondrial complexes I, II, and IV, respectively. Protein samples were harvested after 72 h of treatment at DIV 10 and tested in a 96-well microplate format using a plate reader (Synergy H1, BioTek). Activity was calculated as mΔOD per min, accounting for differences in protein concentration between samples. All reagents listed were obtained from Sigma unless otherwise noted.

UO activity was measured as documented previously (Ma et al., 2011). Samples were added to a reagent containing 25 mM potassium phosphate (pH 7.2) (Cat# P5655), 5 mM MgCl_2_ (Cat# M4880), 1 mM KCN (Cat# 60178), 0.13 mM NADH (Cat# N8129), 65 μM coenzyme Q10 (Cat# C9538), 2.5 mg/ml BSA (Cat# A9418), and 2 μg/ml antimycin A (Cat# A8764). The reagent was heated to 30°C for 10 min before adding 2 ug/ml rotenone (Cat# R8875), followed by adding samples. Activity was tied to reduction of NADH, measured as a decrease in absorbance at 340 nm over a 20-min period.

SDH activity was measured as documented previously (Cimen et al., 2010). Samples were added to a reagent containing 10 mM KCl (Cat# P5405), 5 mM MgCl_2_, 50 mM sodium succinate (Cat# S2378), 40 mM NaN_3_ (Cat# S2002), 300 mM mannitol (Cat# M4125), and 20 mM potassium phosphate (pH 7.2) (all reagents from Sigma). Activity was tied to the reduction of the electron acceptor DPIP (Fisher Chemical, Cat# S286-5) (50 μM), which manifests as a decrease in absorbance at 600 nm over a 30-min period.

COX activity was measured as documented previously (Ma et al., 2011). Samples were added to a reagent containing 20 mM potassium phosphate, pH 7.2, and 0.45 mM n-dodecyl-β-D-maltoside (Sigma, Cat# D4641). Reagent was heated to 30°C for 10 min before adding 15 μM reduced cytochrome C (Sigma, Cat# C2506), followed by adding samples. Activity was tied to oxidation of cytochrome C, measured as a decrease in absorbance at 550 nm over a 30-min period.

### Statistical Analysis

Data were analyzed using Graphpad 8.4.3 software, with the threshold for significance at *p* < 0.05. Values provided are mean ± S.E.M. Student’s *t*-test was used to assess significance between two groups; for other experiments involving high glucose and PGRN, one-way ANOVA was used. *Post hoc* testing was performed using Fisher’s Least Significant Difference (LSD). The *N* and *p* values for experiments are provided in the figure legend or text.

## Results

### Neuronal Morphology Is Promoted Due to Progranulin and Maintained Under High-Glucose Stress

**FIGURE 1:**
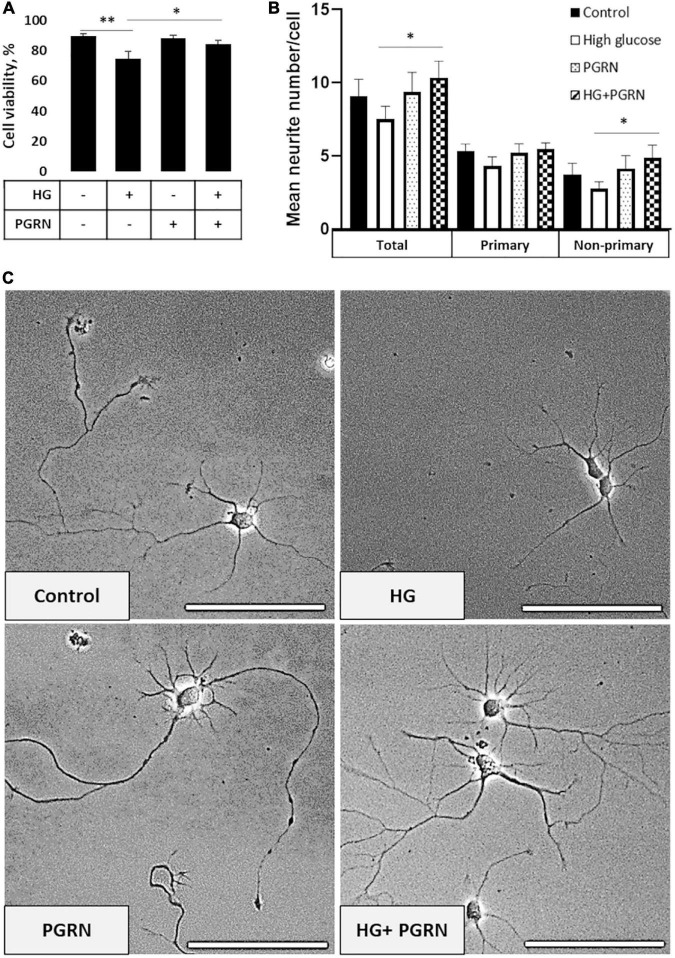
Neuronal viability and neurite outgrowth in neurons treated with high glucose and PGRN. **(A)** Viability of DIV 10 cortical neurons cultured in high-glucose medium (HG) decreased after 72 h from 89.55 ± 1.27% to 74.61 ± 4.80%. PGRN treatment significantly preserved viability under high glucose, with a viability of 84.35 ± 2.29% compared to 74.61 ± 4.80% for HG alone. *N* = 8 fields of view (FoV). **(B)** Number of neurites per cell was counted at DIV 4 after 72 h of treatment using NeuronJ. High glucose lowered mean neurite count from 9.067 ± 1.181 to 7.325 ± 0.784 neurites, while PGRN increased mean neurite count from 7.325 ± 0.784 to 10.353 ± 1.019 neurites. Specifically, this increase was significant in non-primary neurites (2.765 ± 0.481 neurites to 4.882 ± 0.857 neurites) and trending toward significance in primary neurites (4.471 ± 0.438 neurites to 5.471 ± 0.444 neurites). *N* = 13-17 neurons. **(C)** Representative phase-contrast images of primary neurons cultured under high glucose and PGRN after 72 h of treatment. Scale bar, 10 μm. *p < 0.05; **p < 0.01.

To start, we examined neurons to determine if there were any readily noticeable phenotypic differences due to high glucose (HG) or PGRN. Since PGRN is a known neurotrophic and neuroprotective factor (Van Damme et al., 2008), we considered whether this property would be maintained under high-glucose conditions. At DIV 10, cells were treated with 100 mM glucose and 200 ng/ml PGRN for 72 h before testing. This concentration was used in other studies (Chen et al., 2016; Li et al., 2017) and in our case because we saw a significant decrease in cell viability under 100 mM, but not 50 mM, glucose (Supplementary Figure 1). Using a fluorescence-based reporter assay, we found that cell viability decreased significantly (*F* = 5.307, *p* = 0.005) (Figure 1A). Under high-glucose conditions, viability decreased from 89.55 ± 1.27% to 74.61 ± 4.80% (p = 0.001). Despite no difference compared to control (from 89.55 ± 1.27% to 87.94 ± 1.98%, *p* = 0.699), PGRN treatment led to increased viability under high glucose, from 74.61 ± 4.80% to 84.35 ± 2.29% (*p* = 0.025).

Cells viewed under phase-contrast microscopy showed extensive growth of neuritic processes, while cells in high glucose showed less growth of non-primary (i.e., secondary and tertiary) neurites (Figure 1C). Neurite growth appeared to be exceptionally robust with 200 ng/ml PGRN treatment, which was maintained even under high-glucose treatment. While we were unable to perform neurite tracing on matured neurons in culture (>DIV 10) due to the density of cell growth, we were able to assess the effect of PGRN on neurite outgrowth in early developmental (DIV 4) neurons after treatment for 72 h. High glucose incubation resulted in a lower average number of neurites, although this did not reach the threshold of significance (from 9.067 ± 1.181 to 7.325 ± 0.784 neurites, *p* = 0.197) (Figure 1B). PGRN positively influenced neurite outgrowth under high-glucose conditions (from 7.325 ± 0.784 to 10.353 ± 1.019 neurites, *p* = 0.030); when viewed in detail, there was a trend toward an increase in primary neurites (4.471 ± 0.438 to 5.471 ± 0.444 primary neurites, *p* = 0.080), and a significant increase in non-primary neurites (2.765 ± 0.481 to 4.882 ± 0.857 non-primary neurites, *p* = 0.039). This indicates that PGRN treatment promotes neuronal outgrowth and viability, even when cultured in high glucose.

### Primary and HMC3 Cells Show No Change in Progranulin Expression Under High Glucose

**FIGURE 2:**
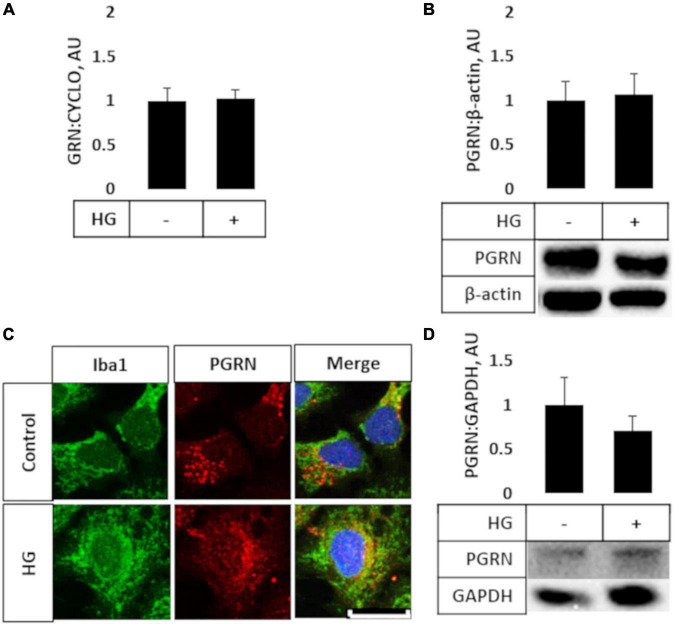
PGRN expression in microglial cells treated with high glucose. HMC3 microglial cells were grown to confluence and treated with high glucose for 72 h before harvest and testing for mRNA **(A)** and protein **(B)** levels of PGRN. A, PGRN mRNA levels were unaffected by high glucose, changing from 1.000 ± 0.139 AU to 1.022 ± 0.099 AU. *N* = 10 samples. **(B)** PGRN protein levels were unaffected by high glucose, changing from 1.000 ± 0.212 AU to 1.069 ± 0.232 AU. *N* = 8 samples. **(C)** Representative immunofluorescence images of primary microglia after 72 h of treatment, with blue as DAPI, green as Iba1, and red as PGRN. Scale bar, 10 μm. **(D)** Western blot analysis of primary cortical cells revealed that PGRN levels were unaffected by glucose concentration, changing from 1.000 ± 0.315 AU to 0.714 ± 0.163 AU. N = 6 samples.

Prior studies have shown that microglia express high levels of PGRN and may be important in neurodegeneration (Mendsaikhan et al., 2019; Choi et al., 2020), so we explored if high glucose affected the degree of PGRN expression in this cell type. We performed qPCR and western blot analyses on HMC3 cells treated with high glucose (in this case, 25 mM, in medium with a basal glucose level of 5.5 mM), and found that mRNA and protein expression of PGRN were similar among control and high glucose-treated cells (Figures 2A,B). The mRNA level changed from 1.000 ± 0.139 Arbitrary Units, AU, to 1.022 ± 0.099 AU, and the protein level from 1.000 ± 0.212 AU to 1.069 ± 0.232 AU. This finding was confirmed in primary cortical cultures, which showed abundant PGRN expression in microglia (Figure 2C) but no difference due to glucose concentration (from 1.000 ± 0.315 AU to 0.714 ± 0.163 AU (Figure 2D). In summary, PGRN expression does not appear to be significantly altered under high glucose, signifying that hyperglycemia-induced neurodegeneration is unlikely due to differential PGRN levels *per se*.

### Progranulin Preserves Light Chain 3B Flux Under High Glucose Conditions

**FIGURE 3:**
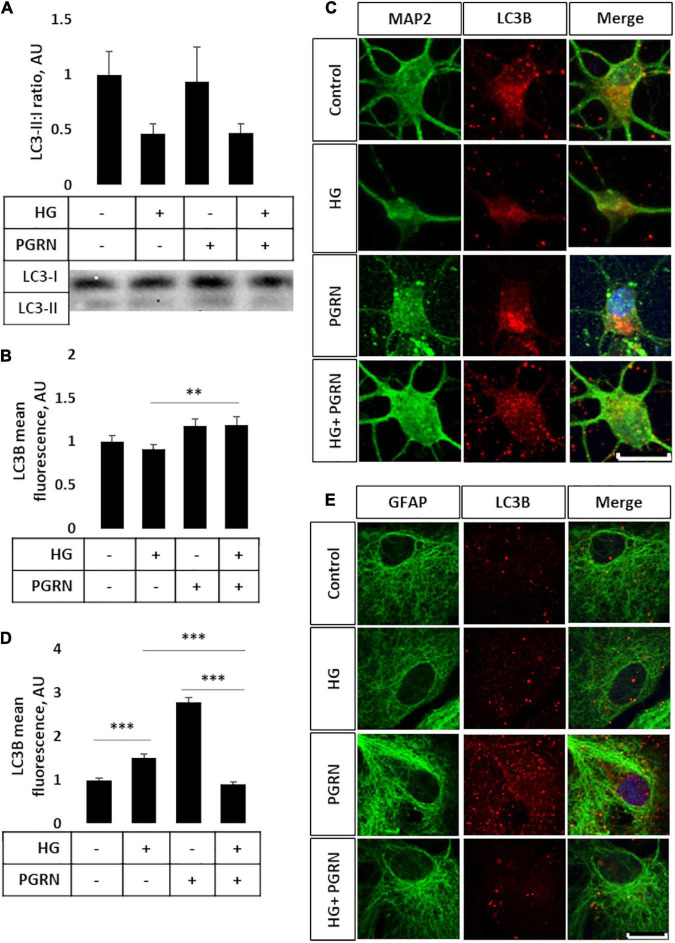
LC3B lipidation and punctate formation in cortical neurons due to high glucose and PGRN. **(A)** Western blot analysis of LC3 lipidation (i.e., LC3-II:I ratio) in primary cortical neurons after 72 h of treatment. The LC3-II:I ratio decreased from control to high glucose (1.000 ± 0.208 AU to 0.464 ± 0.092 AU), an effect that appeared unaffected when treated with PGRN alongside high glucose (0.474 ± 0.076 AU). No difference was observed between control and PGRN-treated samples (0.938 ± 0.309 AU). *N* = 9 samples. **(B)** Immunofluorescence analysis of LC3B puncta expression in neurons after 72 h of treatment. The amount of punctate expression trended toward an increase due to PGRN (from 1.000 ± 0.069 AU, to 1.183 ± 0.077 AU). This difference was significant under high-glucose conditions (from 0.916 ± 0.045 AU, to 1.190 ± 0.097 AU). *N* = 11-29 cells. **(C)** Representative immunofluorescence images of primary neurons after 72 h of treatment, with blue as DAPI, green as MAP2, and red as LC3B. Scale bar, 10 μm. **(D)** Immunofluorescence of LC3B puncta expression in astrocytes after 72 h of treatment increased significantly due to high glucose as well as PGRN treatment (from 1.000 ± 0.0374 AU to 1.512 ± 0.087 AU and 2.792 ± 0.099 AU, respectively). This decreased to control levels under HG + PGRN treatment, to 0.899 ± 0.049 AU. *N* = 12-29 cells. **(E)** Representative immunofluorescence images of primary astrocytes after 72 h of treatment, with blue as DAPI, green as GFAP, and red as LC3B. Scale bar, 10 μm. **p < 0.01; ***p < 0.001.

Impairment of autophagy is seen in diabetes and neurodegenerative conditions, so we tested how high glucose affected autophagy flux using LC3B as an autophagosome marker. Conjugation of LC3 with phosphatidylethanolamine (PE) is an essential step in maturation of the autophagosome, and the rate of autophagy flux can be estimated by measuring the ratio of conjugated to unconjugated protein (i.e., LC3-II:I ratio) via western blot (Mizushima and Yoshimori, 2007). While the overall ANOVA did not reach the threshold of significance (*F* = 2.195, *p* = 0.108), there appears to be a trend toward a decrease in the LC3-II:I ratio due to high glucose compared to control, from 1.000 ± 0.208 AU to 0.464 ± 0.092 AU (Figure 3A). PGRN treatment did not appear to alter LC3B levels in either control (from 1.000 ± 0.208 AU to 0.931 ± 0.309 AU) or high-glucose conditions (to 0.474 ± 0.076 AU). Treatment with the lysosomal inhibitor chloroquine (CQ) led to an increased LC3-II:I ratio (*F* = 5.643, *p* = 0.000), indicative of impaired lysosomal clearance (Supplementary Figure 2A). The change in LC3B was significant in all treatment groups except for PGRN alone (0.940 ± 0.426 AU to 2.147 ± 0.595 AU, *p* = 0.056). Treatment with the autophagy inducer rapamycin also increased LC3B lipidation (F = 2.378, *p* = 0.042), although this was significant only in cells treated with PGRN (0.940 ± 0.426 AU to 7.012 ± 3.711 AU, *p* = 0.006) (Supplementary Figure 2B).

Light chain 3B can also be used to measure autophagosome formation by immunofluorescence, with an increase in punctate formation indicating increased autophagosome formation or decreased autophagosome clearance. We performed immunofluorescence in cortical cells, with LC3B as red and co-stained with either MAP2 as a neuronal marker or GFAP as a glial cell marker (both in green). We found significant changes in LC3B expression in neurons (*F* = 10.45, *p* = 0.001), with a lower (but not significantly so) level of LC3B fluorescence seen when cultured under high-glucose conditions, from 1.000 ± 0.069 AU to 0.916 ± 0.045 AU (*p* = 0.331) (Figure 3B). On the other hand, under high-glucose conditions, PGRN increased puncta levels to 1.190 ± 0.097 AU (*p* = 0.003). It is worth noting that cells labeled with the neuronal marker MAP2 exhibited substantial neurite growth in control conditions, while neurons incubated in high glucose appear to have fewer major primary neurites (Figure 3C), similar to our phase-contrast images (Figure 1C). Differences in LC3B puncta were also pronounced in astrocytes (*F* = 104.6, *p* = 0.000), albeit in the opposite direction, with high glucose significantly increasing LC3B fluorescence in these cells from 1.000 ± 0.037 AU to 1.512 ± 0.087 AU (*p* = 0.001). PGRN increased LC3B intensity even more so, to 2.792 ± 0.099 (*p* = 0.000), but decreasing under high-glucose conditions from 1.512 ± 0.087 AU to 0.899 ± 0.049 AU (*p* = 0.000) (Figures 3D,E). These data on LC3B expression collectively suggest that autophagy flux is decreased under high-glucose conditions and that PGRN alleviates this impairment, with neurons and astrocytes being affected in different ways.

### Lysosomal Turnover Is Promoted by Progranulin Under High-Glucose Conditions Despite Unchanged Lysosome-Associated Membrane Protein 2A Levels

**FIGURE 4:**
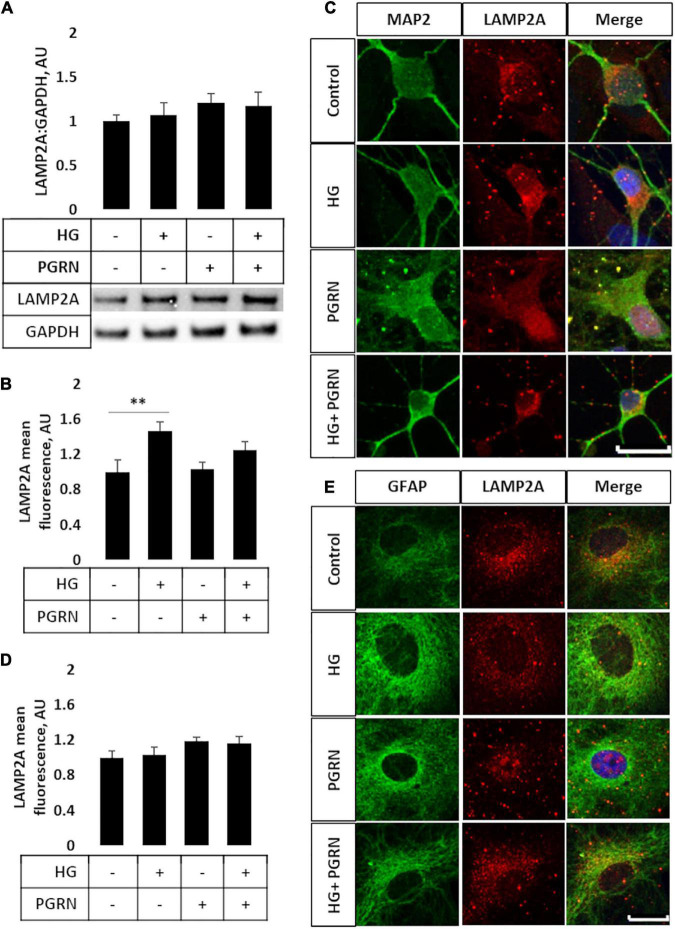
LAMP2A protein levels and punctate localization in cortical neurons and astrocytes due to high glucose and PGRN treatment. **(A)** Total protein LAMP2A levels are unchanged among treatment groups in primary cortical neurons treated for 24 h. *N* = 6 samples. **(B)** Perinuclear LAMP2A punctate formation increased in neurons under HG (from 1.000 ± 0.137 AU to 1.463 ± 0.108 AU), an effect that was attenuated with PGRN (from 1.031 ± 0.083 AU to 1.246 ± 0.096 AU). *N* = 12-27 cells. **(C)** Representative immunofluorescence images of primary neurons after 72 h of treatment, with blue as DAPI, green as MAP2, and red as LAMP2A. Scale bar, 10 μm. **(D)** Perinuclear LAMP2A punctate formation appeared to show a trend toward an increase due to PGRN but was not statistically significant (*F* = 1.825, *p* = 0.151). *N* = 16-22 cells. **(E)** Representative immunofluorescence images of primary astrocytes after 72 h of treatment, with blue as DAPI, green as GFAP, and red as LAMP2A. Scale bar, 10 μm. **p < 0.01.

Later steps of autophagy consist of autophagosome fusion with lysosomal vesicles, which has been shown to be impaired in diabetes (Ma et al., 2017). To see if this step was affected by PGRN, we performed western blot and immunofluorescence studies for LAMP2A, a lysosome membrane protein that localizes in the perinuclear region upon autophagy activation. By western blot, we observed no change in total LAMP2A levels in cells (*F* = 3.271, *p* = 0.621) (Figure 4A) and an increase when cells were treated with the lysosomal inhibitor CQ (*F* = 5.75, *p* = 0.002) and autophagy inducer rapamycin (F = 3.763, p = 0.004) (Supplementary Figures 3A,B). LAMP2A protein levels increased in all groups due to rapamycin and due to CQ except control, which trended toward an increase (1.000 ± 0.074 AU to 2.453 ± 0.497 AU, *p* = 0.094).

However, we observed differential LAMP2A punctate formation in the perinuclear region of cells treated with PGRN. By immunofluorescence, we observed an increase in LAMP2A puncta in neurons (*F* = 4.423, *p* = 0.007), with high glucose increasing punctate levels from 1.000 ± 0.137 AU to 1.463 ± 0.108 AU (*p* = 0.005) (Figures 4B,C). PGRN co-treatment alongside high glucose reduced punctate formation to 1.246 ± 0.096 AU, which was no longer significantly different from control (from 1.000 ± 0.137 AU, *p* = 0.160) or PGRN treatment alone (from 1.031 ± 0.083 AU, *p* = 0.152). In astrocytes, we saw a non-significant trend toward an increase in LAMP2A expression in cells treated with PGRN (*F* = 1.825, *p* = 0.151) (Figures 4D,E). Combined with our data on LC3B, this indicates that lysosome levels are affected by PGRN in cells under high-glucose stress, and that this effect is also different in neurons and astrocytes.

### Turnover of AGEs Is Promoted by Progranulin Under High-Glucose Stress

**FIGURE 5:**
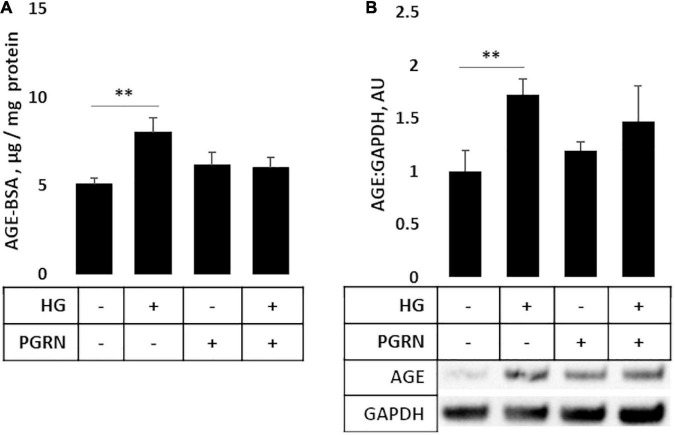
Protein turnover in cortical neurons treated with high glucose and PGRN. **(A)** Cells incubated with high-glucose medium and progranulin for 72 h were treated with 50 μg of AGE-BSA during the remaining 6 h before harvest. Remaining AGE-BSA was measured by fluorescence (360 nm excitation, 460 nm emission) and calculated using a standard curve. High glucose increased AGE-BSA levels from 0.025 ± 0.002 μg/mg protein to 0.040 ± 0.004 μg/mg protein. PGRN was not significantly higher than control (0.031 ± 0.004 μg/mg protein), and no difference was observed due to high glucose alongside PGRN treatment (0.032 ± 0.003 μg/mg protein). *N* = 5-6 samples. **(B)** Western blot analysis of samples in **(A)**. Increased AGE-BSA in high glucose from 1.000 ± 0.197 to 1.726 ± 0.144, indicative of reduced protein turnover, was seen. PGRN prevented the change due to glucose concentration (from 1.198 ± 0.081 to 1.473 ± 0.746) without significantly increasing from control samples (*p* = 0.792). *N* = 5-6 samples. **p < 0.01.

AGEs build up under hyperglycemic conditions as excess glucose spontaneously glycosylates proteins (Singh et al., 2014). Under conditions of impaired autophagy, these modified proteins build up due to a lack of clearance (Takahashi et al., 2017). As a more direct metric of protein turnover, we incubated cortical cells at the end of a 72-h treatment period with 50 μg of AGE-BSA and continued treatment for 6 h at 37°C before harvest. We observed a significant change in AGE levels (*F* = 3.271, *p* = 0.047); in particular, AGE levels were higher in cells under high glucose compared to the control, increasing from 5.067 ± 0.385 μg/mg protein to 8.004 ± 0.852 μg/mg protein (*p* = 0.007) (Figure 5A). While AGE levels were slightly higher in samples treated with 200 ng/ml PGRN than in control at 6.158 ± 0.734 μg/mg protein, this was not significant (p = 0.266), and high glucose did not increase concentration further (up to 6.033 ± 0.575 μg/mg protein; *p* = 0.812 between PGRN and HG + PGRN), suggesting that PGRN treatment aided in clearance of AGE from cells. We also found similar results via western blot (*F* = 3.231, *p* = 0.047), with an overall increase due to high glucose from 1.000 ± 0.196 AU to 1.726 ± 0.150 AU (*p* = 0.009 between control and high glucose). There was no significant increase due to PGRN (to 1.195 ± 0.078 AU) compared to control (*p* = 0.438 between control and PGRN) and high-glucose treatment did not increase this further (to 1.377 ± 0.260; *p* = 0.472 between PGRN and HG + PGRN) (Figure 5B). These data indicate that PGRN may aid in clearance of protein substrates that accumulate due to high-glucose stress.

### Progranulin Modulates Mitochondrial Activity Under High-Glucose Stress in a Complex-Specific Manner

**FIGURE 6:**
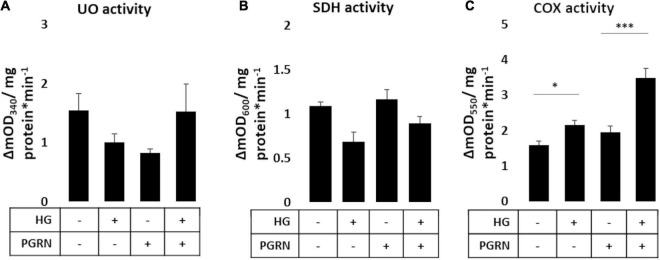
Mitochondrial complex activity in primary cortical neurons in response to high glucose and PGRN. Cells were treated with high glucose and PGRN for 72 h before harvest and enzymatic testing via microplate assay. **(A)** Ubiquinone oxidoreductase (UO) activity was unchanged among treatment conditions. **(B)** Succinate dehydrogenase (SDH) activity decreased under high glucose from 1.138 ± 0.069 ΔmOD_600_ to 0.817 ± 0.172 ΔmOD_600_. While PGRN alone did not alter activity (1.203 ± 0.026 ΔmOD_600_), it attenuated the decrease in activity due to high glucose (1.069 ± 0.129 ΔmOD_600_). N = 6 samples. **(C)** Cytochrome C oxidase (COX) activity was increased by high glucose from 1.596 ± 0.100 ΔmOD_550_ to 2.167 ± 0.122 ΔmOD_550_. With PGRN, high glucose amplified this increase from 1.948 ± 0.183 ΔmOD_550_ to 3.482 ± 0.274 ΔmOD_550_. *N* = 5-6 samples. *p < 0.05; ***p < 0.001.

Mitochondrial activity is dysregulated in diabetes and neurodegenerative diseases like Parkinson’s. Mitochondrial damage is a major contributor of reactive oxygen species (ROS) leakage and cellular dysfunction, and is also seen in hyperglycemic conditions (Rolo and Palmeira, 2006). Because proper maintenance of mitochondrial function through regular turnover is important to neuronal metabolic health (Rugarli and Langer, 2012), we aimed to see if preserving autophagy also improved mitochondrial function. We therefore monitored mitochondrial enzymatic activity as a metric of mitochondrial function, measuring the function of complex I (ubiquinone oxidoreductase, UO), complex II (succinate dehydrogenase, SDH), and complex IV (cytochrome C oxidase, COX). Our results were mixed, with results varying by complex. UO activity, measured in terms of ΔmOD_340_, did not change under any treatment condition (*F* = 1.373, *p* = 0.283) (Figure 6A). SDH activity trended toward significance (*F* = 2.209, *p* = 0.119), with a trend toward a decrease under high-glucose conditions, observed as a decrease in ΔmOD_600_ from 1.138 ± 0.069 to 0.817 ± 0.172 (*p* = 0.060). PGRN treatment attenuated the decrease due to high glucose from 1.203 ± 0.026 to 1.069 ± 0.129 ΔmOD_600_ (*p* = 0.414 between PGRN and HG + PGRN) (Figure 6B). COX activity was significantly altered by high glucose and PGRN (*F* = 20.89, *p* = 0.000), with ΔmOD_550_ increasing under high-glucose conditions from 1.596 ± 0.100 to 2.167 ± 0.122 (*p* = 0.028). PGRN treatment amplified this increase from 1.948 ± 0.183 to 3.482 ± 0.274 (*p* = 0.000 between PGRN and HG + PGRN) despite no change when comparing control to PGRN treatment alone (ΔmOD_550_ from 1.596 ± 0.100 to 1.948 ± 0.183, *p* = 0.156) (Figure 6C). This indicates that the impact of hyperglycemia and PGRN on the mitochondria is complex-specific.

### Extracellular Signal-Regulated Kinase 1/2 and GSK3β Phosphorylation Are Affected by Progranulin Under High-Glucose Conditions

**FIGURE 7:**
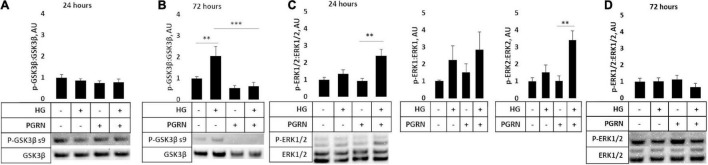
GSK3β and ERK phosphorylation in cortical neurons due to high glucose and PGRN treatment. Cells were treated with high glucose and PGRN for 24 or 72 h before harvest and western blot analysis. **(A,B)** GSK3β phosphorylation at serine nine was unaltered by high glucose or PGRN at 24 h **(A)** but showed significant change at 72 h **(B)**. At 72 h, high glucose increased phosphorylation from 1.000 ± 0.078 AU to 2.044 ± 0.445 AU, while PGRN treatment lowered phosphorylation in both control (1.000 ± 0.078 AU to 0.550 ± 0.112 AU) and high-glucose conditions (2.044 ± 0.445 AU to 0.628 ± 0.178 AU). *N* = 5-6 samples. **(C)** ERK1/2 phosphorylation at threonine 202/tyrosine 204 increased with simultaneous PGRN and high-glucose conditions from 1.000 ± 0.145 AU to 2.403 ± 0.381 AU at 24 h of treatment. Upon closer analysis, ERK2, but not ERK1, phosphorylation was significantly affected, with ERK2 phosphorylation increasing from 1.000 ± 0.197 AU to 3.401 ± 0.544 AU. *N* = 3 samples. **(D)** ERK1/2 phosphorylation at threonine 202/tyrosine 204 was not significantly altered by high glucose or PGRN after 72 h. *N* = 6 samples. **p < 0.01; ***p < 0.001.

Several signaling pathways are implicated in diabetes, including those involved in cell stress such as GSK3β and ERK1/2. The former is implicated in autophagy activation through downstream ULK1 activation (Lin et al., 2012), and the latter has been shown to promote autophagy induction under stressful conditions (Cagnol and Chambard, 2010). We found that PGRN influences the phosphorylation of these kinases in high-glucose conditions, albeit with different timings. Inhibitory GSK3β phosphorylation at serine 9 was not significantly affected after 24 h of treatment (*F* = 0.809, *p* = 0.504) (Figure 7A). After 72 h of treatment, phosphorylation increased significantly (*F* = 7.606, *p* = 0.002), with high glucose increasing phosphorylation from 1.000 ± 0.078 AU to 2.044 ± 0.445 AU (*p* = 0.009) (Figure 7B). PGRN treatment reduced phosphorylation from 1.000 ± 0.078 AU to 0.551 ± 0.112 AU (*p* = 0.221), although a statistically significant decrease was only observed under high-glucose conditions (2.044 ± 0.445 AU to 0.628 ± 0.178 AU, *p* = 0.001). On the other hand, we found that activatory threonine 202 and tyrosine 204 phosphorylation of ERK1/2 significantly increased after 24 h of treatment (*F* = 7.750, *p* = 0.009) (Figure 7C) and returned to baseline within 72 h (*F* = 0.759, *p* = 0.530) (Figure 7D). At 24 h of treatment, it increased under simultaneous high-glucose and PGRN treatment from 1.000 ± 0.145 AU to 2.403 ± 0.381 AU (*p* = 0.004 between control and HG + PGRN). However, high glucose and PGRN treatments alone did not elicit any change, only changing ERK1/2 phosphorylation to 1.342 ± 0.398 and 0.932 ± 0.241 AU, respectively (*p* = 0.352 and 0.848, respectively). This effect appears to affect ERK2 (*F* = 8.436, *p* = 0.007) as well as ERK1 phosphorylation despite a non-significant change in the latter (*F* = 1.206, *p* = 0.368). The lack of significance at 72 h was also present when looking at ERK1 (*F* = 1.784, *p* = 0.183) and ERK2 (*F* = 1.115, *p* = 0.366) in particular. These findings suggest that GSK3β and ERK1/2 activation may play a role in PGRN’s autophagy-modulating response in neurons cultured in high glucose in a time-dependent manner.

## Discussion

In this study, we posit that PGRN may have neuroprotective roles against high glucose-induced reductions in neuronal health and autophagy function. Previous work has connected PGRN to neurodegeneration due to FTLD (Paushter et al., 2018), Alzheimer’s (Minami et al., 2014), and Parkinson’s (Kampen et al., 2014). Research in T2D is mixed, with brain and renal tissues showing a protective effect (Kampen et al., 2014; Zhou et al., 2019b), while in adipose tissue, higher PGRN is deleterious (Zhou et al., 2015). However, no published work to our knowledge has looked at PGRN in the context of hyperglycemia-impaired neuronal autophagy flux and protein turnover. In line with evidence of PGRN’s neuroprotective and growth factor properties, we observed preserved cellular viability (Figure 1A) and neurite outgrowth in cultured neurons (Figures 1B,C) under high-glucose stress.

Microglia express high levels of PGRN (Mendsaikhan et al., 2019), and while studies have explored PGRN levels in brain tissue, those that studied PGRN in the context of T2D examined serum (Youn et al., 2009; Qu et al., 2013) and non-neuronal cell levels (Zhou et al., 2019b). We found that while microglia expressed high levels of PGRN, treatment with high glucose for 72 h did not significantly affect PGRN mRNA or protein expression (Figure 2). This suggests that short-term hyperglycemia does not regulate PGRN levels in brain tissue.

Progranulin has been demonstrated to protect against neurodegeneration through autophagy activation (Paushter et al., 2018; Zhou et al., 2019a), but this has not been explored in the context of hyperglycemia; for that reason, we looked into autophagy flux in response to high glucose. The process of autophagy can be broken down into initiation, nucleation, fusion, and degradation steps, with blockage of each having a distinct signature (Khandia et al., 2019). During nucleation, the light chain protein LC3 is conjugated with PE and attached to both membranes of the developing phagophore, serving as a useful metric of autophagy flux via western blot (Mizushima and Yoshimori, 2007). We observed a decrease in the LC3-II:I ratio in neurons under high glucose, with a minor attenuation due to PGRN (Figure 3A). Treatment of cells with the lysosomal inhibitor CQ resulted in the expected increase in LC3-II:I due to autophagosome buildup, although this increase did not reach the level of significance when treated with PGRN (Supplementary Figure 2A). Rapamycin treatment also increased this ratio, reflective of increased autophagy activation due to mTORC1 inhibition (Li et al., 2014), but this was only significant in cells treated with PGRN (Supplementary Figure 2B). Studies of hyperglycemia in other cell types have shown increased LC3 puncta (Lenoir et al., 2015; Ma et al., 2017; Sakai et al., 2019), so we performed immunofluorescence studies as well. We observed greater LC3 accumulation in PGRN-treated neurons and decreased accumulation in astrocytes under high glucose (Figure 3). Despite no significant change in the LC3-II:I ratio with PGRN under high-glucose conditions, it is possible that the increased LC3 puncta formation is indicative of greater autophagosome maturation due to PGRN.

While total protein levels of the lysosomal membrane protein LAMP2A were unchanged (Figure 4A), we observed increased perinuclear punctate expression of neurons treated with high glucose that was attenuated with PGRN (Figures 4B,C). This, coupled with the increase in LAMP2A protein due to CQ (Supplementary Figure 3A) and seemingly inverse relationship between LAMP2A puncta and autophagy flux (Figure 3A), suggests a buildup of lysosomes in addition to impaired autophagy in neurons under high glucose. PGRN may also play a role in attenuating this, given the reduced buildup of LC3-II when treated alongside CQ (Supplementary Figure 2A). Transfecting cells with dual fluorescence-tagged LC3 (Kimura et al., 2007) could determine if the increased LAMP2A observed represents lysosomes or autolysosomes (the fusion of autophagosome and lysosome).

An unexpected finding in our observations is that LC3B and LAMP2A puncta expression differed in astrocytes and in neurons (Figures 3, 4). Specifically, we observed a marked decrease in LC3B puncta in astrocytes due to PGRN under high glucose (Figure 4D). Glia, particularly astrocytes, play important roles in maintaining neuronal health, including mediating immune responses and regulating glucose metabolism (García-Cáceres et al., 2016; Ortiz-Rodriguez and Arevalo, 2020). While there is less research focusing on their role in autophagy, recent work supports a model in which glia, and astrocytes specifically, contribute to autophagy in neurons (Di Malta et al., 2012; Ortiz-Rodriguez and Arevalo, 2020), possibly through secretion and clearance by surrounding microglia (Choi et al., 2020). Future studies in isolated astrocytic and neuronal fractions are needed to verify if high glucose differentially affects autophagy in these cell types.

While LC3B and LAMP2A are commonly-used markers, there are caveats to consider when interpreting (Mizushima and Yoshimori, 2007; Kaushik and Cuervo, 2009). We therefore sought a more direct metric of protein turnover using AGE-BSA as a substrate. Other studies have monitored protein turnover as a measure of autophagy function using radioisotope and fluorescent reporters (Lui et al., 2016; Orhon and Reggiori, 2017), and our interest in AGE specifically is due to its accumulation under hyperglycemic conditions (Nowotny et al., 2015). Through western blot and microplate assay, we observed increased AGE levels under high-glucose conditions, with PGRN preventing this increase (Figure 5). Alongside our images of LAMP2A punctate formation (Figure 4B), this further supports a model in which proteins targeted for autophagy accumulate under high-glucose conditions, and that PGRN promotes in their breakdown.

Dysfunctional glucose metabolism from hyperglycemia leads to impaired mitochondrial well-being (Sears and Perry, 2015). Defective mitochondria are normally degraded via autophagy as a quality control mechanism (Um and Yun, 2017), which has been shown to be dysregulated under hyperglycemic conditions (Rovira-Llopis et al., 2017). Because prior research has implicated PGRN mutations to impaired mitophagy signaling (Gaweda-Walerych et al., 2021), we investigated if PGRN would aid in preserving mitochondrial function as a downstream consequence of improved mitophagy regulation. Enzymatic studies of mitochondrial function focus on complexes I, II, and IV, so we examined each to obtain a more granular understanding of how high glucose and PGRN affect activity. Our data showed that PGRN and hyperglycemia affect mitochondrial enzyme activity in a complex-specific manner (Figure 6), which may be due to the difference in reaction dynamics involved between enzymes. Interestingly, we found that the decrease in SDH activity due to high glucose was prevented by PGRN (Figure 6B), and that the increase in COX activity due to high glucose was potentiated by PGRN despite no change due to PGRN alone (Figure 6C). Hyperglycemic cell stress increases intracellular Ca^2+^ levels in the cytosol of neurons (Pereira et al., 2010), an effect that has been shown to contribute to de-phosphorylation of an inhibitory site on COX (Ramzan et al., 2021). Since uncontrolled mitochondrial activity leads to excess ROS generation and subsequent cell death, it is possible that PGRN’s neuroprotective effects enable cells under high-glucose conditions to sustain increased COX activity with less deleterious outcomes.

Hyperglycemia activates pathways that both upregulate and downregulate autophagy, and it is the balance between them that determines the overall trajectory of the cell. For instance, cell stressors like high glucose can activate autophagy through ERK activation (Cagnol and Chambard, 2010), although hyperglycemia also inhibits it through mTORC1 activation and subsequent GSK3β inhibition (Kim et al., 2011; Muriach et al., 2014). Because of this, we investigated the role of ERK1/2 and GSK3β. We found that activatory ERK1/2 phosphorylation increased in neurons treated with high glucose and PGRN simultaneously after 24 h (Figure 7C). Similar time-course results were also observed in the hypothalamus of diabetic mouse models treated with Fibroblast Growth Factor 1 (Brown et al., 2021). This activation of ERK1/2 may also contribute to increased mitochondrial activity, as its activation is implicated in proper mitophagy function (Lei et al., 2018; Liu et al., 2021). Interestingly, GSK3β phosphorylation was unchanged at 24 h, but decreased significantly with simultaneous high glucose and PGRN treatment after 72 h (Figure 7B). This could suggest some interplay between the two kinases, as GSK3β has been demonstrated to prevent nuclear localization of ERK1/2, a downstream effect of the latter’s activation (Ma et al., 2008). GSK3β has also been implicated in autophagy activation through the GSK3β-TIP60-ULK1 pathway, so this reduced phosphorylation may also reflect autophagy induction (Nie et al., 2016).

Diabetes is a widespread disease that results in impaired autophagy, protein buildup, and neurodegeneration. Hyperglycemia specifically contributes to pathology in the nervous system, and in the search for a mechanistic cause, multiple pathways have been implicated. Dysfunction in the final steps of autophagy, fusion, and degradation via lysosomes, has been postulated as the cause behind hyperglycemic impairment of autophagy (Nixon et al., 2008; Ma et al., 2017). While PGRN has been a candidate of interest in neurodegeneration and diabetes (Nicoletto and Canani, 2015; Paushter et al., 2018), there has been a lack of research tying PGRN, diabetes-induced neurodegeneration, and autophagy together. We found that PGRN may alleviate high-glucose pathology through upregulation of autophagy, and that it also seems to preserve mitochondrial function under high-glucose conditions. Furthermore, our studies indicate that ERK signaling may also play a role in PGRN’s mechanism of action. However, further research in diabetic cell and animal models needs to be performed to verify PGRN’s neuroprotective role against diabetic stress.

## Data Availability Statement

The raw data supporting the conclusions of this article will be made available by the authors, without undue reservation.

## Ethics Statement

The animal study was reviewed and approved by the Saint Louis University Animal Care and Use Committee.

## Author Contributions

CD, AN, and FX conceptualized and planned the experimental study. CD, VM, and GA performed experiments and performed data analysis. CD, VM, GA, AN, and FX prepared and edited the manuscript.

## Conflict of Interest

The authors declare that the research was conducted in the absence of any commercial or financial relationships that could be construed as a potential conflict of interest.

## Publisher’s Note

All claims expressed in this article are solely those of the authors and do not necessarily represent those of their affiliated organizations, or those of the publisher, the editors and the reviewers. Any product that may be evaluated in this article, or claim that may be made by its manufacturer, is not guaranteed or endorsed by the publisher.

## References

[B1] Almeida M. R. Macário M. C. Ramos L. Baldeiras I. Ribeiro M. H. Santana I. (2016). Portuguese family with the co-occurrence of frontotemporal lobar degeneration and neuronal ceroid lipofuscinosis phenotypes due to progranulin gene mutation. *Neurobiol. Aging* 41 .e1–.e200. 10.1016/j.neurobiolaging.2016.02.019 27021778

[B2] Baker M. Mackenzie I. R. Pickering-Brown S. M. Gass J. Rademakers R. Lindholm C. (2006). Mutations in progranulin cause tau-negative frontotemporal dementia linked to chromosome 17. *Nature* 442 916–919. 10.1038/nature05016 16862116

[B3] Brown J. M. Bentsen M. A. Rausch D. M. Phan B. A. Wieck D. Wasanwala H. (2021). Role of hypothalamic MAPK/ERK signaling and central action of FGF1 in diabetes remission. *IScience* 24:102944. 10.1016/j.isci.2021.102944 34430821 PMC8368994

[B4] Cagnol S. Chambard J.-C. (2010). ERK and cell death: mechanisms of ERK-induced cell death - apoptosis, autophagy and senescence: ERK and cell death. *FEBS J.* 277 2–21. 10.1111/j.1742-4658.2009.07366.x 19843174

[B5] Cairns N. J. Neumann M. Bigio E. H. Holm I. E. Troost D. Hatanpaa K. J. (2007). TDP-43 in Familial and Sporadic Frontotemporal Lobar Degeneration with Ubiquitin Inclusions. *Am. J. Pathol.* 171 227–240. 10.2353/ajpath.2007.070182 17591968 PMC1941578

[B6] Chang S.-C. Yang W.-C. V. (2016). Hyperglycemia, tumorigenesis, and chronic inflammation. *Crit. Rev. Oncol. Hematol.* 108 146–153. 10.1016/j.critrevonc.2016.11.003 27931833

[B7] Chen M. Zheng H. Wei T. Wang D. Xia H. Zhao L. (2016). High Glucose-Induced PC12 Cell Death by Increasing Glutamate Production and Decreasing Methyl Group Metabolism. *BioMed. Res. Internat.* 2016:4125731. 10.1155/2016/4125731 27413747 PMC4930799

[B8] Choi I. Zhang Y. Seegobin S. P. Pruvost M. Wang Q. Purtell K. (2020). Microglia clear neuron-released α-synuclein via selective autophagy and prevent neurodegeneration. *Nat. Comm.* 11:1386. 10.1038/s41467-020-15119-w 32170061 PMC7069981

[B9] Cimen H. Han M.-J. Yang Y. Tong Q. Koc H. Koc E. C. (2010). Regulation of Succinate Dehydrogenase Activity by SIRT3 in Mammalian Mitochondria. *Biochemistry* 49 304–311. 10.1021/bi901627u 20000467 PMC2826167

[B10] Cruts M. Gijselinck I. van der Zee J. Engelborghs S. Wils H. Pirici D. (2006). Null mutations in progranulin cause ubiquitin-positive frontotemporal dementia linked to chromosome 17q21. *Nature* 442 920–924. 10.1038/nature05017 16862115

[B11] Denton D. Kumar S. (2019). Autophagy-dependent cell death. *Cell Death Diff.* 26 605–616. 10.1038/s41418-018-0252-y 30568239 PMC6460387

[B12] Di Malta C. Fryer J. D. Settembre C. Ballabio A. (2012). Astrocyte dysfunction triggers neurodegeneration in a lysosomal storage disorder. *Proc. Natl. Acad. Sci.* 109 E2334–E2342. 10.1073/pnas.1209577109 22826245 PMC3435187

[B13] García-Cáceres C. Quarta C. Varela L. Gao Y. Gruber T. Legutko B. (2016). Astrocytic Insulin Signaling Couples Brain Glucose Uptake with Nutrient Availability. *Cell* 166 867–880. 10.1016/j.cell.2016.07.028 27518562 PMC8961449

[B14] Gass J. Cannon A. Mackenzie I. R. Boeve B. Baker M. Adamson J. (2006). Mutations in progranulin are a major cause of ubiquitin-positive frontotemporal lobar degeneration. *Hum. Mol. Genet.* 15 2988–3001. 10.1093/hmg/ddl241 16950801

[B15] Gaweda-Walerych K. Walerych D. Berdyñski M. Buratti E. Zekanowski C. (2021). Parkin Levels Decrease in Fibroblasts With Progranulin (PGRN) Pathogenic Variants and in a Cellular Model of PGRN Deficiency. *Front. Mol. Neurosci.* 14:676478. 10.3389/fnmol.2021.676478 34054428 PMC8155584

[B16] Kampen J. M. V. Baranowski D. Kay D. G. (2014). Progranulin Gene Delivery Protects Dopaminergic Neurons in a Mouse Model of Parkinson’s Disease. *PLoS One* 9:e97032. 10.1371/journal.pone.0097032 24804730 PMC4013129

[B17] Kaushik S. Cuervo A. M. (2009). Methods to Monitor Chaperone-Mediated Autophagy. *Methods Enzymol.* 452 297–324. 10.1016/S0076-6879(08)03619-7 19200890 PMC4300957

[B18] Khandia R. Dadar M. Munjal A. Dhama K. Karthik K. Tiwari R. (2019). A Comprehensive Review of Autophagy and Its Various Roles in Infectious, Non-Infectious, and Lifestyle Diseases: current Knowledge and Prospects for Disease Prevention, Novel Drug Design, and Therapy. *Cells* 8:674. 10.3390/cells8070674 31277291 PMC6678135

[B19] Kim J. Kundu M. Viollet B. Guan K.-L. (2011). AMPK and mTOR regulate autophagy through direct phosphorylation of Ulk1. *Nat. Cell Biol.* 13 132–141. 10.1038/ncb2152 21258367 PMC3987946

[B20] Kimura S. Noda T. Yoshimori T. (2007). Dissection of the Autophagosome Maturation Process by a Novel Reporter Protein, Tandem Fluorescent-Tagged LC3. *Autophagy* 3 452–460. 10.4161/auto.4451 17534139

[B72] Lei Q. Tan J. Yi S. Wu N. Wang Y. Wu H. (2018). Mitochonic acid 5 activates the MAPK-ERK-yap signaling pathways to protect mouse microglial BV-2 cells against TNFα-induced apoptosis via increased Bnip3-related mitophagy. *Cell. Mol. Biol. Lett*. 23:14. 10.1186/s11658-018-0081-5 29636771 PMC5887257

[B21] Lenoir O. Jasiek M. Hénique C. Guyonnet L. Hartleben B. Bork T. (2015). Endothelial cell and podocyte autophagy synergistically protect from diabetes-induced glomerulosclerosis. *Autophagy* 11 1130–1145. 10.1080/15548627.2015.1049799 26039325 PMC4590611

[B22] Li J. Kim S. G. Blenis J. (2014). Rapamycin: One Drug, Many Effects. *Cell Metab.* 19 373–379. 10.1016/j.cmet.2014.01.001 24508508 PMC3972801

[B23] Li Y. Zhang Y. Wang L. Wang P. Xue Y. Li X. (2017). Autophagy impairment mediated by S-nitrosation of ATG4B leads to neurotoxicity in response to hyperglycemia. *Autophagy* 13 1145–1160. 10.1080/15548627.2017.1320467 28633005 PMC5529069

[B24] Lin S.-Y. Li T. Y. Liu Q. Zhang C. Li X. Chen Y. (2012). GSK3-TIP60-ULK1 Signaling Pathway Links Growth Factor Deprivation to Autophagy. *Science* 336 477–481. 10.1126/science.1217032 22539723

[B73] Liu H. Ho P. W.-L. Leung C.-T. Pang S. Y.-Y. Chang E. E. S. Choi Z. Y.-K. (2021). Aberrant mitochondrial morphology and function associated with impaired mitophagy and DNM1L-MAPK/ERK signaling are found in aged mutant Parkinsonian LRRK2R1441G mice. *Autophagy* 17, 3196–3220. 10.1080/15548627.2020.1850008 33300446 PMC8526027

[B25] Liu J. Li L. (2019). Targeting Autophagy for the Treatment of Alzheimer’s Disease: challenges and Opportunities. *Front. Mol. Neurosci.* 12:203. 10.3389/fnmol.2019.00203 31507373 PMC6713911

[B26] Lui H. Zhang J. Makinson S. R. Cahill M. K. Kelley K. W. Huang H.-Y. (2016). Progranulin Deficiency Promotes Circuit-Specific Synaptic Pruning by Microglia via Complement Activation. *Cell* 165 921–935. 10.1016/j.cell.2016.04.001 27114033 PMC4860138

[B27] Ma C. Bower K. A. Chen G. Shi X. Ke Z. J. Luo J. (2008). Interaction between ERK and GSK3beta mediates basic fibroblast growth factor-induced apoptosis in SK-N-MC neuroblastoma cells. *J. Biol. Chem.* 283 9248–9256. 10.1074/jbc.M707316200 18263590 PMC2431019

[B28] Ma L.-Y. Lv Y.-L. Huo K. Liu J. Shang S.-H. Fei Y.-L. (2017). Autophagy-lysosome dysfunction is involved in Aβ deposition in STZ-induced diabetic rats. *Behav. Brain Res.* 320 484–493. 10.1016/j.bbr.2016.10.031 27773683

[B29] Ma Y.-Y. Zhang X.-L. Wu T.-F. Liu Y.-P. Wang Q. Zhang Y. (2011). Analysis of the Mitochondrial Complex I-V Enzyme Activities of Peripheral Leukocytes in Oxidative Phosphorylation Disorders. *J. Child Neurol.* 26 974–979. 10.1177/0883073811399905 21540367

[B30] Madhusudhanan J. Suresh G. Devanathan V. (2020). Neurodegeneration in type 2 diabetes: alzheimer’s as a case study. *Brain Behav.* 10:5. 10.1002/brb3.1577 32170854 PMC7218246

[B31] Martens L. H. Zhang J. Barmada S. J. Zhou P. Kamiya S. Sun B. (2012). Progranulin deficiency promotes neuroinflammation and neuron loss following toxin-induced injury. *J. Clin. Invest.* 2012:163113. 10.1172/JCI63113 23041626 PMC3484443

[B32] Mendsaikhan A. Tooyama I. Walker D. G. (2019). Microglial Progranulin: Involvement in Alzheimer’s Disease and Neurodegenerative Diseases. *Cells* 8:230. 10.3390/cells8030230 30862089 PMC6468562

[B33] Minami S. S. Min S.-W. Krabbe G. Wang C. Zhou Y. Asgarov R. (2014). Progranulin protects against amyloid β deposition and toxicity in Alzheimer’s disease mouse models. *Nat. Med.* 20 1157–1164. 10.1038/nm.3672 25261995 PMC4196723

[B34] Mir S. U. R. George N. M. Zahoor L. Harms R. Guinn Z. Sarvetnick N. E. (2015). Inhibition of Autophagic Turnover in β-Cells by Fatty Acids and Glucose Leads to Apoptotic Cell Death. *J. Biolog. Chem.* 290 6071–6085. 10.1074/jbc.M114.605345 25548282 PMC4358249

[B35] Mizushima N. Klionsky D. J. (2007). Protein Turnover Via Autophagy: implications for Metabolism. *Annu. Rev. Nutrit.* 27 19–40. 10.1146/annurev.nutr.27.061406.093749 17311494

[B36] Mizushima N. Yoshimori T. (2007). How to Interpret LC3 Immunoblotting. *Autophagy* 3 542–545. 10.4161/auto.4600 17611390

[B37] Moruno F. Pérez-Jiménez E. Knecht E. (2012). Regulation of Autophagy by Glucose in Mammalian Cells. *Cells* 1 372–395. 10.3390/cells1030372 24710481 PMC3901114

[B38] Muriach M. Flores-Bellver M. Romero F. J. Barcia J. M. (2014). Diabetes and the Brain: oxidative Stress, Inflammation, and Autophagy. *Oxid. Med. Cell. Long.* 2014 1–9. 10.1155/2014/102158 25215171 PMC4158559

[B39] Nguyen A. D. Nguyen T. A. Martens L. H. Mitic L. L. Farese R. V. (2013b). Progranulin: at the interface of neurodegenerative and metabolic diseases. *Trends Endocrinol. Metab.* 24 597–606. 10.1016/j.tem.2013.08.003 24035620 PMC3842380

[B40] Nguyen A. D. Nguyen T. A. Cenik B. Yu G. Herz J. Walther T. C. (2013a). Secreted progranulin is a homodimer and is not a component of high density lipoproteins (HDL). *J. Biol. Chem.* 288 8627–8635. 10.1074/jbc.m112.441949 23364791 PMC3605681

[B41] Nicoletto B. B. Canani L. H. (2015). The role of progranulin in diabetes and kidney disease. *Diabet. Metab. Syndr.* 7:117. 10.1186/s13098-015-0112-6 26697121 PMC4687133

[B42] Nie T. Yang S. Ma H. Zhang L. Lu F. Tao K. (2016). Regulation of ER stress-induced autophagy by GSK3β-TIP60-ULK1 pathway. *Cell Death Dis.* 7 e2563–e2563. 10.1038/cddis.2016.423 28032867 PMC5260977

[B43] Nixon R. A. Yang D.-S. Lee J.-H. (2008). Neurodegenerative lysosomal disorders: a continuum from development to late age. *Autophagy* 4 590–599. 10.4161/auto.6259 18497567

[B44] Nowotny K. Jung T. Höhn A. Weber D. Grune T. (2015). Advanced Glycation End Products and Oxidative Stress in Type 2 Diabetes Mellitus. *Biomolecules* 5 194–222. 10.3390/biom5010194 25786107 PMC4384119

[B45] Ogata M. Hino S.-I. Saito A. Morikawa K. Kondo S. Kanemoto S. (2006). Autophagy Is Activated for Cell Survival after Endoplasmic Reticulum Stress. *Mol. Cell. Biol.* 26 9220–9231. 10.1128/MCB.01453-06 17030611 PMC1698520

[B46] Orhon I. Reggiori F. (2017). Assays to Monitor Autophagy Progression in Cell Cultures. *Cells* 6:20. 10.3390/cells6030020 28686195 PMC5617966

[B47] Ortiz-Rodriguez A. Arevalo M.-A. (2020). The Contribution of Astrocyte Autophagy to Systemic Metabolism. *Internat. J. Mol. Sci.* 21:2479. 10.3390/ijms21072479 32260050 PMC7177973

[B48] Pagano G. Polychronis S. Wilson H. Giordano B. Ferrara N. Niccolini F. (2018). Diabetes mellitus and Parkinson disease. *Neurology* 90 e1654–e1662. 10.1212/WNL.0000000000005475 29626177

[B49] Paushter D. H. Du H. Feng T. Hu F. (2018). The lysosomal function of progranulin, a guardian against neurodegeneration. *Acta Neuropathologica* 136 1–17. 10.1007/s00401-018-1861-8 29744576 PMC6117207

[B50] Pereira T. de O. S. da Costa G. N. F. Santiago A. R. S. Ambrósio A. F. dos Santos P. F. M. (2010). High glucose enhances intracellular Ca2+ responses triggered by purinergic stimulation in retinal neurons and microglia. *Brain Res.* 1316 129–138. 10.1016/j.brainres.2009.12.034 20034478

[B51] Perry D. C. Lehmann M. Yokoyama J. S. (2013). Progranulin Mutations as Risk Factors for Alzheimer Disease. *JAMA Neurol.* 70 774–778. 10.1001/2013.jamaneurol.393 23609919 PMC3743672

[B52] Qu H. Deng H. Hu Z. (2013). Plasma Progranulin Concentrations Are Increased in Patients with Type 2 Diabetes and Obesity and Correlated with Insulin Resistance. *Mediat. Inflamm.* 2013:360190. 10.1155/2013/360190 23476101 PMC3588183

[B53] Ramzan R. Kadenbach B. Vogt S. (2021). Multiple Mechanisms Regulate Eukaryotic Cytochrome C Oxidase. *Cells* 10:514. 10.3390/cells10030514 33671025 PMC7997345

[B54] Rolo A. P. Palmeira C. M. (2006). Diabetes and mitochondrial function: role of hyperglycemia and oxidative stress. *Toxicol. Appl. Pharm.* 212 167–178. 10.1016/j.taap.2006.01.003 16490224

[B55] Ross C. A. Poirier M. A. (2004). Protein aggregation and neurodegenerative disease. *Nat. Med.* 10 S10–S17. 10.1038/nm1066 15272267

[B56] Rovira-Llopis S. Bañuls C. Diaz-Morales N. Hernandez-Mijares A. Rocha M. Victor V. M. (2017). Mitochondrial dynamics in type 2 diabetes: pathophysiological implications. *Redox Biol.* 11 637–645. 10.1016/j.redox.2017.01.013 28131082 PMC5284490

[B57] Rugarli E. I. Langer T. (2012). Mitochondrial quality control: a matter of life and death for neurons: mitochondrial quality control and neurodegeneration. *EMBO J.* 31 1336–1349. 10.1038/emboj.2012.38 22354038 PMC3321185

[B58] Sakai S. Yamamoto T. Takabatake Y. Takahashi A. Namba-Hamano T. Minami S. (2019). Proximal Tubule Autophagy Differs in Type 1 and 2 Diabetes. *J. Am. Soc. Nephrol.* 30 929–945. 10.1681/ASN.2018100983 31040190 PMC6551771

[B59] Sears B. Perry M. (2015). The role of fatty acids in insulin resistance. *Lipids Health Dis.* 14:121. 10.1186/s12944-015-0123-1 26415887 PMC4587882

[B60] Sergi D. Renaud J. Simola N. Martinoli M.-G. (2019). Diabetes, a Contemporary Risk for Parkinson’s Disease: epidemiological and Cellular Evidences. *Front. Aging Neurosci.* 11:302. 10.3389/fnagi.2019.00302 31787891 PMC6856011

[B61] Singh V. P. Bali A. Singh N. Jaggi A. S. (2014). Advanced Glycation End Products and Diabetic Complications. *Korean J. Physiol. Pharm.* 18 1–14. 10.4196/kjpp.2014.18.1.1 24634591 PMC3951818

[B62] Smith K. R. Damiano J. Franceschetti S. Carpenter S. Canafoglia L. Morbin M. (2012). Strikingly different clinicopathological phenotypes determined by progranulin-mutation dosage. *Am. J. Hum. Genet.* 90 1102–1107. 10.1016/j.ajhg.2012.04.021 22608501 PMC3370276

[B63] Stavoe A. K. H. Holzbaur E. L. F. (2019). Autophagy in Neurons. *Annu. Rev. Cell Dev. Biol.* 35 477–500. 10.1146/annurev-cellbio-100818-125242 31340124 PMC6996145

[B64] Takahashi A. Takabatake Y. Kimura T. Maejima I. Namba T. Yamamoto T. (2017). Autophagy Inhibits the Accumulation of Advanced Glycation End Products by Promoting Lysosomal Biogenesis and Function in the Kidney Proximal Tubules. *Diabetes* 66 1359–1372. 10.2337/db16-0397 28246295

[B65] Um J.-H. Yun J. (2017). Emerging role of mitophagy in human diseases and physiology. *BMB Rep.* 50 299–307. 10.5483/BMBRep.2017.50.6.056 28366191 PMC5498140

[B66] Van Damme P. Van Hoecke A. Lambrechts D. Vanacker P. Bogaert E. van Swieten J. (2008). Progranulin functions as a neurotrophic factor to regulate neurite outgrowth and enhance neuronal survival. *J. Cell Biol.* 181 37–41. 10.1083/jcb.200712039 18378771 PMC2287280

[B67] Van Kampen J. M. Kay D. G. (2017). Progranulin gene delivery reduces plaque burden and synaptic atrophy in a mouse model of Alzheimer’s disease. *PLo*S *One* 12:e0182896. 10.1371/journal.pone.0182896 28837568 PMC5570501

[B68] Youn B.-S. Bang S.-I. Kloting N. Park J. W. Lee N. Oh J.-E. (2009). Serum Progranulin Concentrations May Be Associated With Macrophage Infiltration Into Omental Adipose Tissue. *Diabetes* 58 627–636. 10.2337/db08-1147 19056610 PMC2646061

[B69] Zhou B. Li H. Liu J. Xu L. Guo Q. Sun H. (2015). Progranulin induces adipose insulin resistance and autophagic imbalance via TNFR1 in mice. *J. Mol. Endocrinol.* 55 231–243. 10.1530/JME-15-0075 26373796

[B71] Zhou D. Zhou M. Wang Z. Fu Y. Jia M. Wang X. (2019a). Progranulin alleviates podocyte injury via regulating CAMKK/AMPK-mediated autophagy under diabetic conditions. *J. Mol. Med.* 97 1507–1520. 10.1007/s00109-019-01828-3 31402399

[B70] Zhou D. Zhou M. Wang Z. Fu Y. Jia M. Wang X. (2019b). PGRN acts as a novel regulator of mitochondrial homeostasis by facilitating mitophagy and mitochondrial biogenesis to prevent podocyte injury in diabetic nephropathy. *Cell Death Dis.* 10:524. 10.1038/s41419-019-1754-3 31285425 PMC6614416

