# Supplementary material for: Progranulin Preserves Autophagy Flux and Mitochondrial Function in Rat Cortical Neurons Under High Glucose Stress
Source: Front Cell Neurosci. 2022 Jul 8;16:874258. doi: 10.3389/fncel.2022.874258 (PMC9308004; doi:10.3389/fncel.2022.874258)
Supplement: Supplementary file 1 [file Data_Sheet_1.pdf]

Supplementary Material

1 Supplementary Data

Supplementary Material should be uploaded separately on submission. Please include any supplementary data, figures and/or tables. All supplementary files are deposited to FigShare for permanent storage and receive a DOI.

Supplementary material is not typeset so please ensure that all information is clearly presented, the appropriate caption is included in the file and not in the manuscript, and that the style conforms to the rest of the article. To avoid discrepancies between the published article and the supplementary material, please do not add the title, author list, affiliations or correspondence in the supplementary files.

2 Supplementary Figures and Tables

For more information on Supplementary Material and for details on the different file types accepted, please see here. Figures, tables, and images will be published under a Creative Commons CC-BY licence and permission must be obtained for use of copyrighted material from other sources (including re-published/adapted/modified/partial figures and images from the internet). It is the responsibility of the authors to acquire the licenses, to follow any citation instructions requested by third-party rights holders, and cover any supplementary charges.

2.1 Supplementary Figures

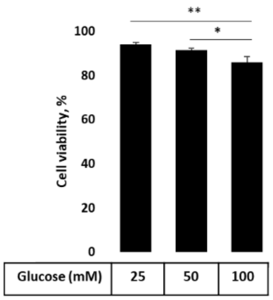

Supplementary Figure 1. Cell viability in neurons treated under different glucose concentrations. Primary cortical neurons were incubated in Neurobasal medium containing either 25 mM (control), 50 mM, or 100 mM glucose. After 72 hours of treatment, viability showed a dose-dependent decrease as glucose concentration increased. Viability decreased from 94.18±0.62% to 91.32±0.98% for 50 mM glucose and to 85.87±2.50% for 100 mM glucose. N=7-9 FoV. *, $p<0.05$; **, $p<0.01$.

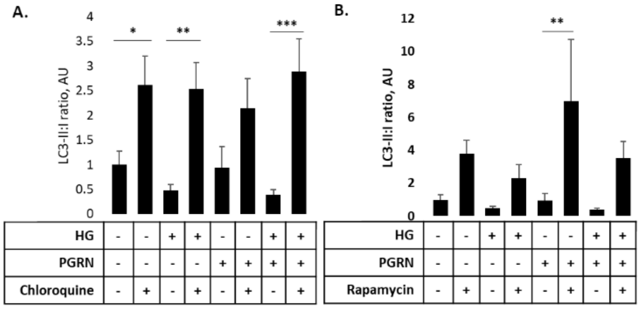

Supplementary Figure 2. LC3B lipidation in cortical neurons under positive and negative regulators. Western blot analysis of LC3 lipidation was performed in primary cortical neurons treated with high glucose and PGRN for 72 hours. Cells were additionally treated with either the lysosomal inhibitor chloroquine (CQ, A) for the final 6 hours of treatment, or with the autophagy inducer rapamycin (B) for 72 hours alongside HG and PGRN treatment. A, LC3 lipidation increased due to CQ treatment impaired autophagosome breakdown, resulting in elevated LC3-II:I ratio. This was significant among most treatment groups (control: 1.000±0.279 AU to 2.616±0.580 AU; HG: 0.480±0.118 AU to 2.543±0.523 AU; HG+PGRN: 0.396±0.096 AU to 2.887±0.668 AU). CQ elevated LC3-II:I under PGRN treatment alone, although to a lesser extent (0.940±0.426 AU to 2.147±0.595 AU). There was no difference in LC3-II:I among all groups treated with CQ. N=5-6 samples. B, LC3 lipidation increased under all treatment groups (control: 1.000±0.279 AU to 3.802±0.828 AU; HG: 0.480±0.118 AU to 2.299±0.824 AU; HG+PGRN: 0.396±0.096 AU to 3.527±1.017 AU), but was only statistically significant in cells treated with PGRN alone (0.940±0.426 AU to 7.012±3.711 AU, p=0.006). N=4-6 samples. *, p<0.05; **, p<0.01; ***, p<0.001.

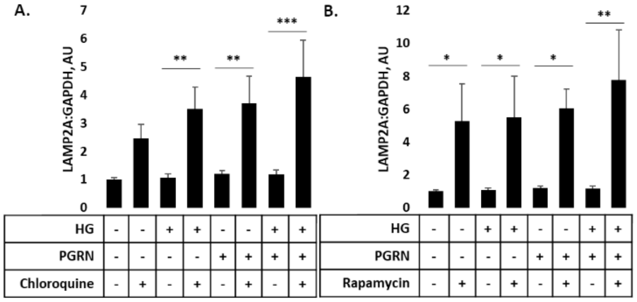

Supplementary Figure 3. LAMP2A expression in cortical neurons under positive and negative regulators. Western blot analysis of LAMP2A levels was performed in primary cortical neurons treated with high glucose and PGRN for 72 hours. Cells were additionally treated with either the lysosomal inhibitor chloroquine (CQ, A) for the final 6 hours of treatment, or with the autophagy inducer rapamycin (B) for 72 hours alongside HG and PGRN treatment. A, LAMP2A protein expression increased due to CQ, suggesting increased lysosomal buildup. The increase due to CQ was significant in all treatment groups (HG: 1.077±0.137 AU to 3.503±0.768 AU, PGRN: 1.210±0.108 AU to 3.714±0.949 AU, HG+PGRN: 1.176±0.158 AU to 4.640±1.295 AU) except control, which trended towards significance (1.000±0.074 AU to 2.453±0.497 AU, p=0.094). N=5-6 samples. B, LAMP2A protein expression increased due to rapamycin among all treatment groups (control: 1.000±0.074 AU to 5.287±2.266 AU; HG: 1.077±0.137 AU to 5.488±2.531 AU; PGRN:

1.210±0.108 AU to 6.035±1.178 AU; HG+PGRN: 1.176±0.158 AU to 7.765±3.058 AU). N=4-6 samples. *, $p<0.05$; **, $p<0.01$; ***, $p<0.001$.
